# ATAD2 Overexpression Identifies Colorectal Cancer Patients with Poor Prognosis and Drives Proliferation of Cancer Cells

**DOI:** 10.1155/2015/936564

**Published:** 2015-11-30

**Authors:** Yang Luo, Guang-Yao Ye, Shao-Lan Qin, Min-Hao Yu, Yi-Fei Mu, Ming Zhong

**Affiliations:** Department of Gastrointestinal Surgery, Renji Hospital, School of Medicine, Shanghai Jiao Tong University, Shanghai 200127, China

## Abstract

ATPase family AAA domain-containing 2 (ATAD2) has been identified as a critical modulator involved in cell proliferation and invasion. The purpose of this study was to explore the expression of ATAD2 in CRC tissues as well as its relationship with degree of malignancy. Data containing three independent investigations from Oncomine database demonstrated that ATAD2 is overexpressed in CRC compared with normal tissue, and similar result was also found in 32 pairs of CRC tissues by qPCR. The protein expression of ATAD2 was examined in six CRC cell lines and 300 CRC specimens. The results showed that high expression of ATAD2 was significantly correlated with tumor size (*P* < 0.001), serum CEA (*P* = 0.012), lymph node metastasis (*P* = 0.018), liver metastasis (*P* = 0.025), and clinical stage (*P* = 0.004). Kaplan-Meier method suggested that higher ATAD2 protein expression significantly associated with the overall survival (OS) of CRC patients (*P* < 0.001) and was an independent predictor of poor OS. Functional studies showed that suppression of ATAD2 expression with siRNA could significantly inhibit the growth in SW480 and HCT116 cells. These results indicated that ATAD2 could serve as a prognostic marker and a therapeutic target for CRC.

## 1. Introduction

Colorectal cancer (CRC) is one of the most common lethal malignancies in terms of both incidence and mortality [1]. Although the diagnosis and treatment of CRC have been improved, the efficacy of surgery and chemotherapy remains unsatisfactory due to late diagnosis [2]. Therefore, new diagnostic and treatment strategies are urgently needed for this malignancy.

ATPase family AAA domain-containing 2 (ATAD2), also known as ANCCA (AAA+ nuclear coregulator cancer associated), is a novel member of the AAA+ ATPase family [3, 4]. ATAD2 contains both a bromodomain and an ATPase domain and also maps to chromosome 8q24 that is the most commonly amplified region in many types of cancer [5]. The especial structure of ATAD2 indicates that it is associated with genome regulation, including cell proliferation, division, apoptosis, and differentiation [6–10]. Recently, it is reported that aberrant expression of ATAD2 contributes to hepatocellular carcinoma proliferation and metastasis [11, 12]. Studies have revealed that ATAD2 is highly expressed in several types of tumors such as breast cancer, lung cancer, and gastric cancer [13–15]. Thus, ATAD2 manifests oncogenic function and plays a significant role in cancer development. However, the expression of the ATAD2 protein in CRC and its significance remain uncertain.

## 2. Methods

### 2.1. In Silico Analysis Using the Oncomine Database

To determine the expression pattern of ATAD2 in CRC, three datasets (Kaiser Colon, Hong Colorectal, and Hong Colorectal) in Oncomine database (https://www.oncomine.org) were used. We compared ATAD2 gene expression in CRC tissues with normal colorectal tissues according to the standard procedures as previously described [16].

### 2.2. Cell Culture and Transfection

Five human CRC cell lines (HCT116, SW480, LoVo, T84, and HT29) and a normal control colon cell line (HCoEpiC) were all preserved in Shanghai Cancer Institute. All of these cells lines were cultured in DMEM medium (Invitrogen) supplemented with 10% (v/v) fetal bovine serum (FBS) and 1% antibiotics at 37°C in a humidified incubator under 5% CO_2_ condition.

The transfections were performed using Lipofectamine 2000 (Invitrogen, USA). Small interfering RNAs (siRNA) targeting ATAD2 and a negative control were obtained from GenePharma Technology (Shanghai, China). The transfection was performed according to the manufacturer's protocol.

### 2.3. Patients and Tissue Samples

A total of 300 formalin-fixed paraffin-embedded CRC tissues were collected to perform immunohistochemical staining from January 2005 to November 2014 at the Renji Hospital, Shanghai Jiao Tong University School of Medicine, China. Moreover, additional 32 snap-frozen CRC tissues and corresponding adjacent noncancerous tissues to isolate RNA, which were also obtained from Renji Hospital, were enrolled in this study simultaneously. Inclusion criteria were histologically confirmed CRC and curative resection of tumor without preoperative or postoperative adjuvant therapy. Important clinical data, such as tumor location, serum CEA level, and clinical stage, were collected from each patient's medical records. The follow-up time was calculated from the date of surgery to the date of death, or the last known follow-up. All CRC tissue samples in this study were obtained with patients' written informed consent and all experiments have been approved by the ethics committee at local hospital.

### 2.4. Real-Time Quantitative PCR

Total RNA from primary tumor and adjacent noncancerous tissue samples was extracted using Trizol reagent (Takara, Japan), and according to the manufacturer's instructions, reverse transcription was performed by PrimeScript RT-PCR kit (Takara, Japan). Real-time quantitative PCR (qPCR) was performed using a 7500 real-time PCR system (Applied Biosystems, Inc., USA). The primers for ATAD2 were as follows: forward: 5′-GGAATCCCAAACCACTGGACA-3′; reverse: 5′-GGTAGCGTCGTCGTAAAGCACA-3′. GAPDH mRNA was used to standardize the relative expression of ATAD2. The primers for GAPDH were as follows: forward: 5′-GCATTGCCCTCAACGACCAC-3′, reverse: 5′-CCACCACCCTGTTGCTGTAG-3′.

### 2.5. Immunohistochemical Staining

Four-micrometer-thick tissue sections were subjected to immunohistochemical staining with avidin-biotin-peroxidase complex system which was performed as previously described [17]. Tissue sections were incubated by anti-ATAD2 antibody (1 : 400, Abcam, Cambridge, UK) at 4°C overnight. Immunohistochemical staining was scored by two independent pathologists according to intensity and percentage of positive cells simultaneously. Staining intensity was scored as follows: 0: negative; 1: weak staining; 2: moderate staining; 3: strong staining, and the percentage of positive cells was scored on a scale of 0–4 (0, <5%; 1, 5%–30%; 2, 30%–50%; 3, 51%–75%; 4, >75%). And the final score was designated as low or high expression group using the percent of positive cell score × staining intensity score as follows: low expression was defined as a total score < 6 and high expression with a total score ≥ 6.

### 2.6. Western Blot

Western blot was performed as previously described [18]. Cells were harvested after 48-hour transfection, and 40 *μ*g of protein samples was resolved by SDS-PAGE and transferred to PVDF membranes. The membranes were probed with the same primary antibody used in immunostaining overnight at 4°C and incubated for 1 hour with secondary antibody (Boston, MA, USA). The Western blotting analysis was repeated at least three times.

### 2.7. Cell Proliferation Assay

Cell viability was assessed by the Cell Counting Kit 8 (CCK-8; Dojindo). Briefly, control and treated SW480 and HCT116 cells were seeded into 96-well plates at an initial density of 3000 cells/well. At each time point, 10 *μ*L of CCK-8 solution was added to each well and incubated for 2 hours. The absorbance was measured by scanning with a microplate reader at 450 nm. The experiment was repeated at least three times.

### 2.8. Statistical Analysis

Statistical analyses were performed by SPSS 19.0 (SPSS Inc.; Chicago, USA). The expression of ATAD2 mRNA in CRC tissues and corresponding noncancerous tissues was analyzed with the Student's *t*-test. The differences of the relative absorbance value of CCK8 assays also were determined using Student's *t*-test. The Chi-square test was used to analyze the relationship between ATAD2 expression and clinicopathological features. Survival rate was evaluated by Kaplan-Meier method and differences between survival curves were tested by the log-rank test. *P* values less than 0.05 were considered to be statistically significant.

## 3. Results

### 3.1. ATAD2 Is Overexpressed in CRC at mRNA Level

**Figure 1:**
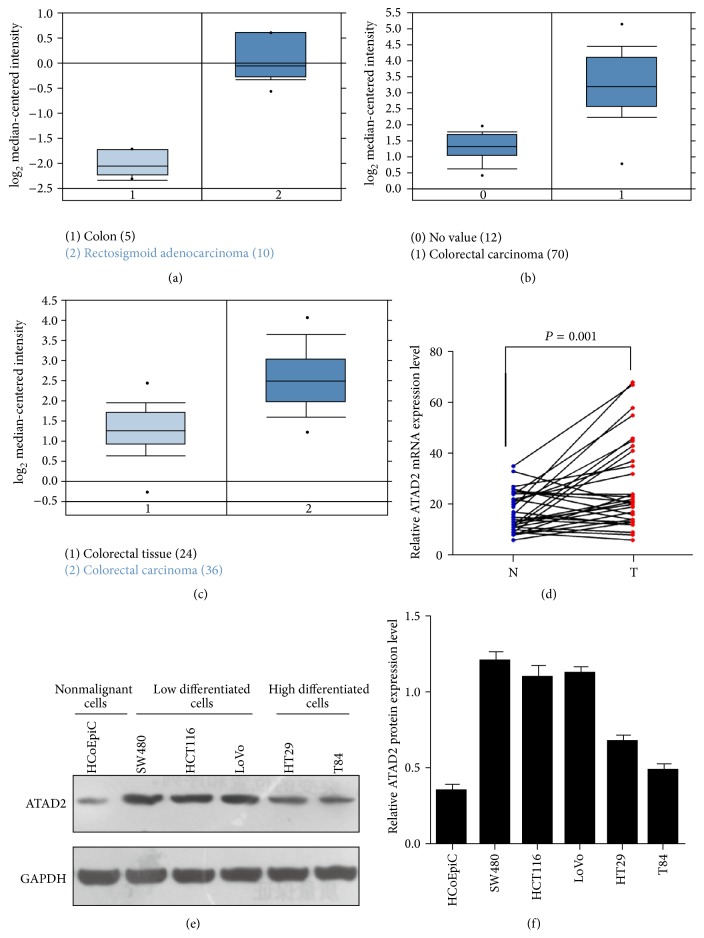
ATAD2 expression in CRC at mRNA and protein level. (a)–(c) ATAD2 expression in Kaiser Colon (a), Hong Colorectal, (b) and Skrzypczak Colorectal (c). (d) Increased ATAD2 mRNA expression in 32 matched tumor (T) and nontumor tissues (N) was detected by qPCR. (e)-(f) Western blots show the ATAD2 expression in five CRC cell lines and the nonmalignant HCoEpiC cells. *P* values were calculated by paired *t*-test.

To roundly investigate ATAD2 expression in CRC, we analyzed three independent microarray datasets from Oncomine database [19–21]. The results showed that the mRNA expression levels of ATAD2 were upregulated in most of CRC tissues, compared with normal tissue (Figures 1(a)–1(c)). Then, 32 pairs of CRC and matched normal tissues were subjected to qPCR. Consistent with the data from Oncomine database, ATAD2 was also overexpressed in 68.75% (22/32) of CRC patients at mRNA level (Figure 1(d)).

### 3.2. ATAD2 Is Expressed Diversely in CRC at Protein Level

**Figure 2:**
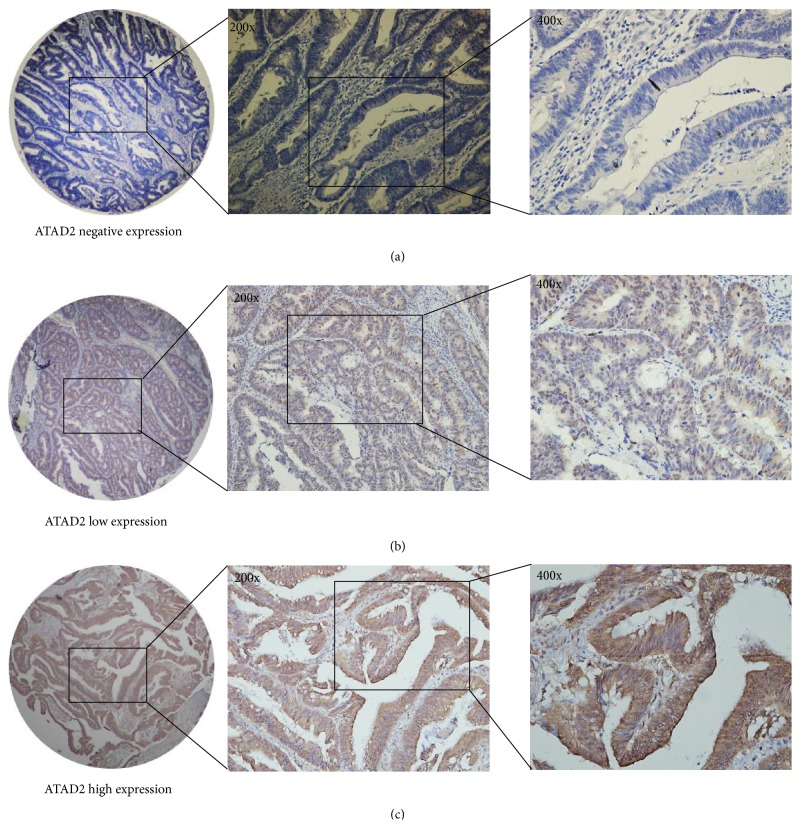
ATAD2 expression in CRC was determined by immunochemistry. (a) Negative expression of ATAD2, (b) low expression of ATAD2, and (c) high expression of ATAD2. Representative images are shown at 200x and 400x magnification, respectively.

To further investigate the expression of ATAD2 at the protein level, we measured ATAD2 level in CRC cell lines and tissues. The expression of ATAD2 protein was increased in all five CRC cell lines compared with the nonmalignant HCoEpiC cells. Moreover, we found that the higher expression of ATAD2 protein is in low differentiated CRC cell lines (SW480, HCT116, and LoVo) compared with well-differentiated cell lines (HT29, T84) (Figures 1(e)-1(f)). Then, we tested 300 CRC tissue samples by using the method of immunohistochemical staining and found that ATAD2 was low expressed in the 124 (41.33%) of the total 300 CRC samples while the remaining 176 (58.67%) samples remained at a high expression level (Figures 2(a)–2(c)).

### 3.3. Relationship between ATAD2 Expression and Corresponding Clinical Parameters

To evaluate the clinical significance of ATAD2, the Chi-square test was used to analyze correlations between ATAD2 protein expression and clinicopathological parameters in CRC. The results indicated that high expression of ATAD2 in CRC tissues is closely correlated with tumor size (*P* < 0.001), serum CEA level (*P* = 0.012), lymph node metastasis (*P* = 0.018), liver metastasis (*P* = 0.025), and clinical stage (*P* = 0.004). However, no statistically significant correlations were identified between ATAD2 expression and other clinicopathologic characteristics, including age, gender, and tumor location (Table 1).

### 3.4. Correlation between ATAD2 Expression and Prognosis in CRC Patients

**Figure 3:**
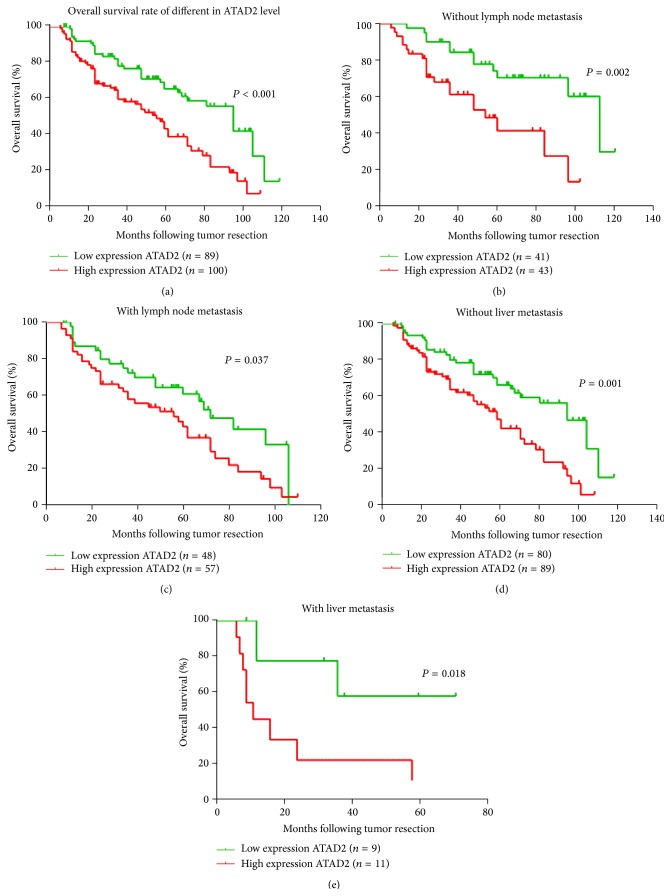
ATAD2 is correlated with overall survival rate in CRC patients. (a) Kaplan-Meier survival curves show high expression level of ATAD2 is significantly correlated with poor survival of CRC. (b)–(e) Correlation between ATAD2 expression and patient survival in colorectal cancer is independent of lymphatic metastasis and liver metastasis. *P* values were calculated using the log-rank test.

To investigate the prognostic influence of ATAD2, the overall survival (OS) rate of CRC patients was analyzed using Kaplan-Meier survival curves and the log-rank test. The result revealed that high expression of ATAD2 was inversely associated with OS for all 189 samples (*P* < 0.001) (Figure 3(a)). In addition, The OS of ATAD2 negative group was distinctly better than that of the ATAD2 positive one for samples separated according to lymphatic metastasis and liver metastasis (Figures 3(b)–3(e)).

Furthermore, univariate and multivariate analyses were performed to confirm the possibility of ATAD2 used as an independent risk factor for poor prognosis in the 182 cases of CRC. Univariate Cox regression analyses showed that ATAD2 expression, tumor size, serum CEA, liver metastasis, and clinical stage were significantly associated with overall survival (OS) (Table 2). The multivariate Cox regression analysis confirmed ATAD2 expression, tumor size, and clinical stage as independent predictors of the OS in CRC patients (Table 2).

### 3.5. Effect of ATAD2 on CRC Cell Proliferation In Vitro

**Figure 4:**
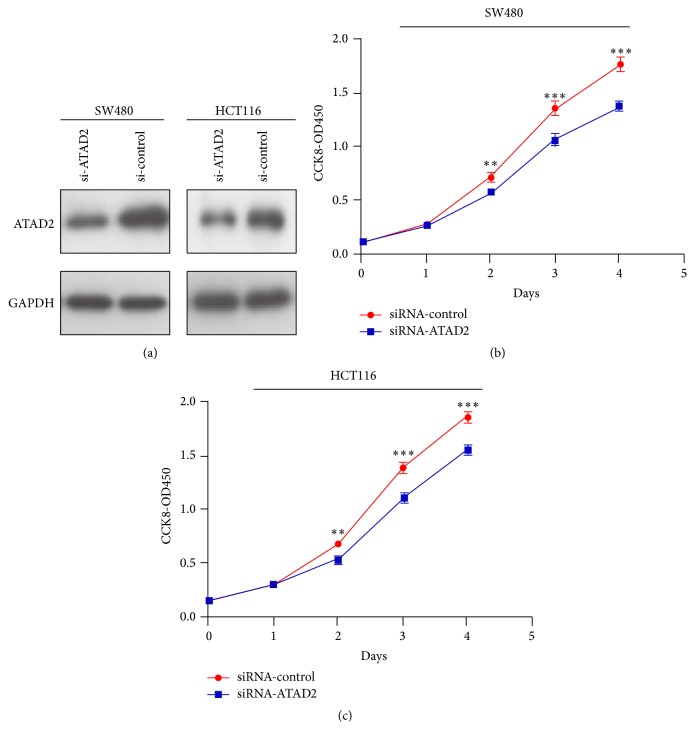
Effect of siRNA-ATAD2 on the proliferation of SW480 and HCT116 cells. (a) ATAD2 knockdown efficiency was confirmed by qPCR in SW480 and HCT116 cells. (b)-(c) siRNA-ATAD2 decreased cell viability measured by CCK8 assays. *P* values were calculated by Student's *t*-test (^*∗∗*^ *P* < 0.01, ^*∗∗∗*^ *P* < 0.001) and the experiment was repeated at least three times.

To better understand the biological function of ATAD2, we transfected siRNAs-ATAD2 into SW480 and HCT116 cells, and the expression levels of endogenous ATAD2 proteins were significantly suppressed by Western blot (Figure 4(a)). Moreover, we found that knockdown of ATAD2 resulted in a significant decrease in cell viability measured by CCK-8, compared with control (Figures 4(b)-4(c)).

## 4. Discussion

Studies focused on human clinical specimens and genetically engineered mouse models of CRC have led to a better understanding of this genetic malignancy [22]. ATAD2 through different mechanisms broadly participated in various tumor types, such as prostate cancer, lung cancer, breast cancer, and ovarian cancer [4, 13, 14, 23]. Previous studies indicated that ATAD2 directly interacted with the oncogene AIB1/ACTR and played an important role in the recruitment of ER*α* to promote the expression of genes driving cancer cell proliferation [24]. Furthermore, the ATPase domain of ATAD2 also enhanced the E2 induction of cyclin D1 and E2F1 expression [4]. In addition, ATAD2 was also directly associated with AR to activate AR-mediated transcription and was required to regulate the expression of androgen-induced genes that controlled cancer cell proliferation and survival [4].

In the current study, we firstly assessed ATAD2 expression at mRNA level. Both data from Oncomine database and our results showed that most primary CRC tissues exhibited significantly higher mRNA expression of ATAD2 than their matched normal tissues. This tendency was then confirmed by immunohistochemistry where 176 of 300 (58.67%) CRC tissues were found to have high protein expression of ATAD2. Previous studies had demonstrated that overexpression of ATAD2 at the transcriptional level via miR-372 regulation promotes tumor malignancy and consequently results in poor outcome in liver adenocarcinoma patients [11]. Our results revealed similar phenomenon that ATAD2 has positive correlation with serum CEA level, lymph node metastasis, distant metastasis, and clinical stage in CRC. Besides, patients with a high level of ATAD2 expression had significantly shorter survival times compared to those with a low level of ATAD2 expression. Furthermore, univariate analysis showed that elevated ATAD2 expression was significantly associated with the OS of CRC patients; multivariate analysis demonstrated that ATAD2 expression, tumor size, and clinical stage are independent risk factors for the prognosis of CRC patients. Collectively, these results suggest that ATAD2 may be involved in the initiation and progression of CRC. Finally, we found that downexpression of ATAD2 could dramatically suppress the proliferation of SW480 and HCT116 cells, which is in keeping with the previous studies on the other cancer cells. However, its underlying molecular mechanisms need to be further investigated.

In conclusion, our study demonstrated that ATAD2 expression was increased in CRC tissues compared to adjacent normal tissues and might be associated with pathological development of CRC. In addition, we found that high expression of ATAD2 could serve as an independent prognostic factor for CRC patients. Therefore, ATAD2 may be an important clinical marker of therapy for CRC.

## Figures and Tables

**Table 1:** Relationship between ATAD2 expression and clinicopathological features in 300 colorectal cancer patients.

Variable	ATAD (*n*)		
	Low	High	*P*
	*n* = 124	*n* = 176	
Age			
≤65 years	73 (24.33)	97 (32.33)	0.518
>65 years	51 (17.00)	79 (26.34)	
Gender			
Male	68 (22.37)	99 (32.57)	0.978
Female	56 (18.42)	81 (26.64)	
Tumor size			
≤5 cm	78 (26.00)	72 (24.00)	**<0.001**
>5 cm	46 (15.33)	104 (34.67)	
Tumor location			
Rectum	73 (24.33)	103 (34.33)	0.873
Colon	51 (17.01)	73 (24.33)	
Serum CEA			
≤5 ng/mL	74 (24.67)	79 (26.33)	**0.012**
>5 ng/mL	50 (16.67)	97 (32.33)	
Lymph node metastasis			
N0	53 (17.67)	52 (17.33)	**0.018**
N1-N2	71 (23.67)	124 (41.33)	
Liver metastasis			
M0	109 (36.33)	137 (45.66)	**0.025**
M1	15 (5.00)	39 (13.00)	
Clinical stage			
I	17 (5.67)	12 (4.00)	**0.004**
II	23 (7.67)	16 (5.33)	
III	69 (23.00)	109 (36.33)	
IV	15 (5.00)	39 (13.00)	

**Table 2:** Univariate and multivariate analyses showing the overall survival in colorectal cancer.

Prognostic parameter	Univariate analysis			Multivariate analysis		
	HR	95% CI	*P* value	HR	95% CI	*P* value
ATAD2 (high versus low)	2.272	1.479–3.491	**0.000**	1.762	1.113–2.790	**0.016**
Age (>65 versus ≤65)	1.393	0.911–2.132	0.127	—	—	—
Gender (male versus female)	1.362	0.905–2.048	0.139	—	—	—
Tumor size (>5 cm versus ≤5 cm)	1.842	1.201–2.825	**0.005**	1.698	1.075–2.681	**0.023**
Tumor location (colon versus rectum)	0.770	0.497–1.194	0.243	—	—	—
Serum CEA (>5 ng/mL versus ≤5 ng/mL)	1.628	1.063–2.493	**0.025**	1.497	0.979–2.291	0.063
Lymph node metastasis (present versus absent)	1.686	1.099–2.588	**0.017**	1.038	0.644–1.673	0.879
Liver metastasis (present versus absent)	3.308	1.777–6.151	**0.000**	1.710	0.777–3.764	0.183
Clinical stage (I vs. II vs. III vs. IV)	1.983	1.549–2.539	**0.000**	1.611	1.168–2.221	**0.004**

HR: hazard ratio; CI: confidence interval. The bold number represents the *P* values with significant differences.
